# First Detection of West Nile Virus Lineage 2 in Mosquitoes in Switzerland, 2022

**DOI:** 10.3390/pathogens12121424

**Published:** 2023-12-07

**Authors:** Stefania Cazzin, Nicole Liechti, Damian Jandrasits, Eleonora Flacio, Christian Beuret, Olivier Engler, Valeria Guidi

**Affiliations:** 1 Institute of Microbiology, Department for Environment Constructions and Design, University of Applied Sciences and Arts of Southern Switzerland (SUPSI), 6850 Mendrisio, Switzerland; damian.jandrasits@supsi.ch (D.J.); eleonora.flacio@supsi.ch (E.F.); valeria.guidi@supsi.ch (V.G.); 2 Spiez Laboratory, Federal Office for Civil Protection, Austrasse, 3700 Spiez, Switzerland; nicole.liechti@babs.admin.ch (N.L.); christian.beuret@babs.admin.ch (C.B.); olivier.engler@babs.admin.ch (O.E.); 3 Graduate School for Cellular and Biomedical Sciences, University of Bern, 3012 Bern, Switzerland

**Keywords:** West Nile virus, arbovirus surveillance, *Culex pipiens*, honey-baited FTA cards

## Abstract

West Nile virus (WNV) is one of the most widespread flaviviruses in the world, and in recent years, it has been frequently present in many Mediterranean and Eastern European countries. A combination of different conditions, such as a favourable climate and higher seasonal average temperatures, probably allowed its introduction and spread to new territories. In Switzerland, autochthonous cases of WNV have never been reported, and the virus was not detected in mosquito vectors until 2022, despite an entomological surveillance in place in Canton Ticino, southern Switzerland, since 2010. In 2022, 12 sites were monitored from July to October, using BOX gravid mosquito traps coupled with honey-baited FTA cards. For the first time, we could detect the presence of WNV in FTA cards and mosquitoes in 8 out of the 12 sampling sites monitored, indicating an unexpectedly widespread circulation of the virus throughout the territory. Positive findings were recorded from the beginning of August until mid-October 2022, and whole genome sequencing analysis identified a lineage 2 virus closely related to strains circulating in Northern Italy. The entomological surveillance has proved useful in identifying viral circulation in advance of possible cases of WNV infection in humans or horses.

## 1. Introduction

West Nile virus (WNV) is an African mosquito-borne virus, belonging to the *Flavivirus* genus, *Flaviviridae* family, within the Japanese encephalitis virus serocomplex [1]. The virus is maintained in the environment through an enzootic cycle involving mosquitoes, mainly of the genus *Culex*, as vectors and birds, serving as amplifying hosts [2]. In addition to wild birds, various mammals and reptiles can also become infected with WNV under natural conditions [3] but usually do not develop levels of viraemia high enough to allow for the virus re-transmission to a competent vector [4]. In most cases, the infection is asymptomatic, but humans and equids may develop a clinical disease, with symptoms characterised by a mild febrile malaise, up to, in a small percentage of cases, fatal meningo-encephalitis [5,6].

West Nile virus was first identified in Uganda in 1937 [7]; subsequently, it was responsible for sporadic cases and disease outbreaks in humans and equids in Europe, the western Mediterranean and Southern Russia in 1962–1964, Belarus and Ukraine in the 1970s and 1980s, Romania in 1996–1997, the Czech Republic in 1997 and Italy in 1998 [2]. It is only since the 1990s that scientific interest in WNV has grown, for its impact on both human and animal health [8]. During the last decade, the virus has shown a progressive expansion into new territories distributed further north, as in the case of Germany [9] or the Netherlands [10]. The ability of WNV to infect numerous mosquito and bird species led to its worldwide spreading. West Nile virus is, nowadays, considered the most widespread flavivirus in the world, currently circulating in Africa, the Middle East, Europe, Western Russia, Southwest Asia, Australia (West Nile virus Kunjin) and the American continent [11].

The Canton Ticino, in the southern part of Switzerland, borders Lombardy and Piedmont, two regions in Northern Italy where WNV was detected in 2013 and 2014, respectively [12,13]. Despite this proximity, Switzerland had never reported the presence of autochthonous cases in humans or animals (equids and birds) [14]. In the Canton Ticino, an active surveillance program for the detection of arboviruses in mosquitoes has been in place since 2010, highlighting the presence of Usutu virus (USUV) in the territory [15,16], but never of the WNV until 2022. Here, we report the first evidence of circulation in Switzerland of WNV lineage 2 in mosquitoes collected during the entomological surveillance carried out in 2022.

## 2. Materials and Methods

### 2.1. Sampling

In 2022, arboviral surveillance on mosquitoes was conducted from the beginning of July to mid-October in twelve sampling sites in the four most southward districts of the Canton of Ticino, southern Switzerland: Mendrisiotto (7 sampling sites), Luganese (2 sampling sites), Locarnese (1 sampling sites), and Bellinzonese (area of the Magadino Plain, 2 sampling sites). All the sites were located in natural and peri-urban environments characterized by the presence of wetland or small lakes and ponds in order to increase the catches of *Culex pipiens* mosquitoes. BOX gravid mosquito traps (BioQuip, Rancho Dominguez, CA, USA) coupled with honey-baited FTA (Flinders Technology Associates) cards [16] and powered by a 6 V, 72 Ah, lead-acid battery were employed in each site. The traps remained in the field for the whole period, and FTA cards and mosquitoes were retrieved from the collection chamber every one or two weeks. Trapped mosquitoes were counted and sorted by species, sex, location, collection date and divided into pools of up to 50 mosquito specimens and stored at −80 °C. For the molecular analysis, only the two main vectors of WNV were considered, *Cx. pipiens* and *Cx. modestus*, even if the diffusion of the second one in the territory was very limited [17]. FTA cards recovered from the traps were stored at −20 °C until analysis for the presence of viral RNA.

### 2.2. Screening of Mosquito Pools and FTA Cards

Viral RNA was extracted from the mosquito pools using the RNeasy Plus Universal kit (Qiagen, Hilden, Germany) according to the manufacturer’s protocol, with slight modifications. Briefly, pools of up to 50 mosquitoes in 300 μL PBS1x were homogenized using a 5 mm stainless-steel bead (Qiagen) in a TissueLyser II device (Qiagen) at 30 Hz for 2 min. An aliquot of 150 μL was then added to 900 μL QIAzol lysis solution and 10 µL Mengovirus vMC0 strain as internal control for the whole process [16], and it was processed according to the manufacturer’s protocol without the addition of the gDNA Eliminator Solution. Final RNA was eluted in 2 × 30 µL RNase-free water.

FTA cards were spiked with Mengovirus vMC0 and eluted with 1 mL QIAcard FTA Wash Buffer (Qiagen). RNA was extracted with the QIAamp Viral RNA Mini Kit (Qiagen), according to the methods described previously [16].

All the samples were analysed for the presence of flaviviruses with semi-nested endpoint RT-PCRs targeting the non-structural protein 5 (NS5) gene of flaviviruses [16,18]. The positive amplified products were then Sanger sequenced for the flavivirus identification [16]. Each positive or doubtful sample was further confirmed by two in-house one-step real-time RT-PCRs: (1) WNV3′ system targeting the 3′ untranslated region (UTR) and allowing for the detection of both WNV and USUV, and (2) WNV5′ system targeting the 5′ UTR, allowing for specific WNV detection (Table 1).

The WNV3′ real-time PCR system consisted of a 20 μL solution containing 1× TaqMan^®^ Fast Virus 1-Step Mastermix (Thermo Fisher Scientific, Waltham, MA, USA), 0.4 μM of forward and reverse primers, 0.25 μM of probe and 4 μL of template RNA. The RT-qPCR reactions were performed in a 7500 Fast Real-Time PCR System (Applied Biosystems, Waltham, MA, USA), with the following thermal cycling conditions: 5 min at 50 °C, 20 s at 95 °C followed by 45 cycles of 3 s at 95 °C and 30 s at 60 °C. The WNV5′ real-time PCR system consisted of a 25 μL solution containing 1× TaqMan^®^ Fast Virus 1-Step Mastermix, 0.4 μM of forward and reverse primers, 0.25 μM of probe and 5 μL of template RNA. The RT-qPCR reactions were performed in a LightCycler 480 Instrument (Roche Diagnostic, Rotkreuz, Switzerland) with the same thermal cycling conditions described above.

### 2.3. Sequencing and Phylogenetic Analysis

Whole genome sequencing of selected RNA samples extracted from FTA cards and mosquitoes was carried out using a WNV specific amplicon panel, as described previously [19,20]. Briefly, RNA samples were reverse transcribed using Superscript IV and amplified using a multiplex PCR in two reaction pools using 35 amplification cycles. A detailed overview of the primers used can be found in Additional File 2 of ref. [20]. Amplified products were pooled and sequenced on a GridION system on R9.4 FLO-MIN106 flow cells using the native barcoding kit (EXP-NBD104) in combination with the genomic sequencing kit (SQK-LSK109). Basecalling was performed using high-accuracy model of guppy v6.0.7 within the MinKNOW software v22.03.4 with barcode trimming activated. Resulting reads were filtered and mapped to the reference AY532665.1 using minimap2 v2.24-r1122 [21] and primers were clipped, and consensus sequences were created using pysam v0.19.1 [22]. Sequences were checked for unexpected frameshifts in homopolymeric regions and manually curated. Sequences have been uploaded to NCBI (OR091151–OR091158).

For phylogenetic analysis, previously sequenced WNV genomes of lineage 2 with origin in Europe were downloaded from NCBI and aligned using MAFFT v.7.505 [23]. A phylogenetic tree was constructed using IQ-Tree v.2.2.0.3 [24], including automatic model selection and 1000 ultrafast bootstrap to obtain branch supports [25]. Tree was rooted with samples of the Russian/Romanian clade as outgroup and visualized using ggtree v3.6.2 [26].

## 3. Results

**Figure 1:**
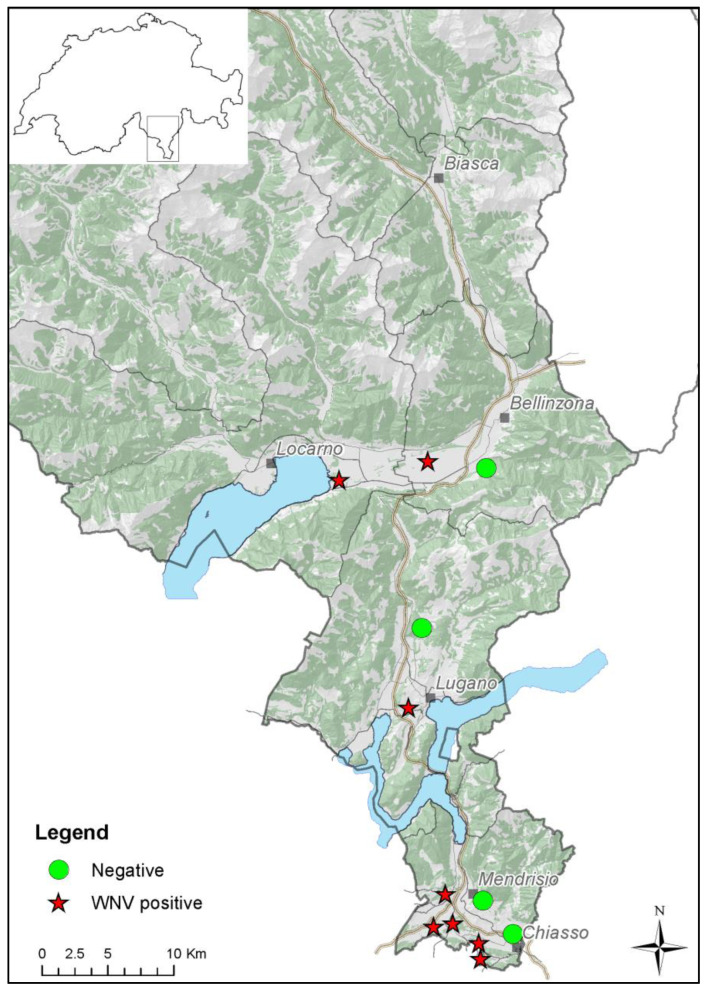
Map of the surveillance areas in Canton Ticino, Switzerland. Red stars: positive sites for WNV; green dots: negative sites. This map was created with ArcGIS software, ArcMap 10.6.1.

After eleven years of arbovirus surveillance in mosquitoes in Ticino, in early August 2022, the first two FTA cards were found positive for West Nile (WNV) in two different sites, one in Bellinzonese district (sampling period: 26 July–8 August) and one in Mendrisiotto district (sampling period: 2 August–9 August). Later, WNV could be detected in six additional sampling sites distributed over all districts in both mosquitoes and FTA cards. Once detected WNV, some of these sampling sites, in the Luganese and the Mendrisiotto districts, remained positive until October 2022 (Figure 1).

In most cases, the positivity was detected in both the cards and the mosquito pools. However, in some cases, the positivity was found either in the FTA card only or in the mosquito pools only, which might be due to the low viral load (Ct value > 35). Sanger sequencing of the semi-nested endpoint RT-PCR products revealed WNV lineage 2 for all sequenced samples. For further characterization, whole genome sequencing of selected RNA samples with Ct values < 32 extracted from FTA cards and mosquitoes was performed using a targeted approach. In total, eight nearly complete genomes could be retrieved (Table 2).

**Figure 2:**
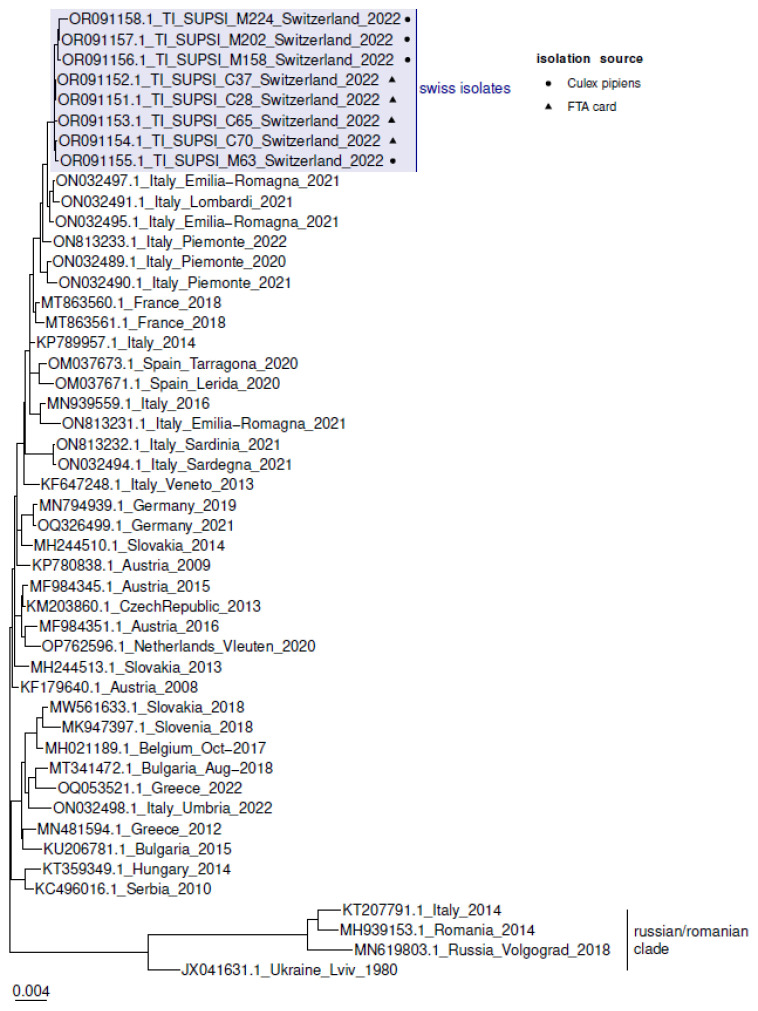
Phylogenetic tree of Swiss West Nile virus lineage 2 isolates. Complete genome sequences of WNV lineage 2 with origin in Europe (n = 40) were included. The maximum-likelihood phylogenetic tree was constructed with IQ-TREE (GTR + F + I + I + R3) and 1000 bootstrap replicates. Tree was rooted using the Russian/Romanian clade as outgroup. The scale bar corresponds to the average number of substitutions per site.

Genome sequences of the different sampling sites have average nucleotide identity between 99.67 and 99.99%. Phylogenetic analysis based on complete genome sequences of WNV lineage 2 with origins in Europe revealed a close relation to strains circulating in Northern Italy in 2021 and 2022 (Figure 2). Isolates between the different sampling sites are highly similar, and no clustering of isolates from the area of the Magadino Plain and the more southern sampling sites Mendrisiotto and Luganese was observed.

## 4. Discussion

The survey of viruses in arthropod vectors is a very important aspect for public health, as it allows both the early detection of possible circulating pathogens and the evaluation of the seasonality and intensity of their circulation, enabling possible preventive measures [27].

The year 2022 was characterized by a high number of WNV outbreaks in Europe [28,29,30], with 965 human cases reported in EU/EEA countries, of which 586 were in Italy; this number is comparable to that observed in the peak epidemic year, 2018 [31]. In Switzerland, no autochthonous cases have been reported in humans or animals [14], not even during the years with the highest viral circulation in Europe, such as 2018 and 2022.

For Switzerland, 2022 was the warmest year by far and, locally, also the sunniest since measures began in 1864. The climatic conditions were characterized by a mild winter and spring and continued during the summer and autumn with higher average temperatures [32]. These conditions favoured the proliferation of the vector *Cx. pipiens* and the establishment of the WNV cycle in natural areas for the first time. The territory of Canton Ticino is characterised by the presence of numerous natural areas with wetlands, lakes and ponds frequented by many species of wild birds and mosquitoes. It is possible that the marked drought conditions recorded in 2022 may have favoured the establishment of the natural cycle of WNV transmission. Drought could, in fact, increase the possibility of contact between wild birds and mosquitoes around the few available water sources, allowing the WNV cycle to take place more rapidly, spreading to both populations [33,34]. The USUV, a flavivirus with a similar enzootic cycle to WNV, is surveyed in mosquitoes and has circulated in Switzerland for several years [15,16,35].

The detection of the WNV in mosquitoes in Switzerland increases the risk of autochthonous animal or human cases. In most cases, however, the infection proceeds asymptomatically and, for this reason, it is possible that some cases have not been identified. So far, the regular monitoring and control plan in place in the urban areas of Canton Ticino since 2000 for the containment of invasive mosquito species such as *Aedes albopictus* [36,37] may also have acted effectively against *Cx. pipiens*, decreasing their density and protecting citizens. In addition, in natural areas, the presence of *Cx. pipiens* may be reduced through treatment programs based on the biopesticide *Bacillus thuringiensis* var. *israelensis* (Bti, VectoBac^®^ G, Valent BioSciences Corp., Libertyville, IL, USA), carried out in spring–early summer in some important wetlands and natural ponds to limit the development of floodwater mosquitoes, especially after heavy rainfall events.

In a One Health approach, mosquito surveillance is very important because it allows for the early detection of the circulation of the virus prior to human and equine cases [38,39,40]. The use of Box Gravid Mosquito traps coupled with honey-baited FTA cards definitely proved to be a very effective system for detecting the circulation of WNV and USUV in a rapid way. The analysis of FTA cards enables the optimization of time and costs, avoiding time-consuming mosquito analysis, including the entomological identification, counting and sorting of mosquitoes under cold conditions, and the processing of several mosquito pools per trap. This system can, therefore, be useful both for areas with low risk of WNV circulation and endemic ones.

## Figures and Tables

**Table 1:** Oligonucleotide primers and probes used for West Nile virus detection by real-time PCR.

Primer	Genome Position ^1^	Sequence (5′–3′) ^2^	Product Size (bp)
LSWNV3’F	10,459–10,478	CGCCACCGGAAGTTGAGTAG	111
LSWNV3’R	10,569–10,550	CTCCTTCCGAGACGGTTCTG	
LSWNV3’P	10,483–10,503	TGCTGCCTGCGDCTCAACCCC	
LSWNV5’F	7–27	TCGCCTGTGTGAGCTGACAAA	112
LSWNV5’R	118–96	GCCCTCCTGGTTTCTTAGACATC	
LSWNV5’P	89–66	TTCGTGCYAAGAAACAGCTCGCAC	

^1^ Positions based on the complete sequence from WNV 956 (GenBank, acc. no. NC_001563). ^2^ Probes are labelled with the fluorescent FAM (6-carboxyfluorescein) reporter dye at the 5′ end and with the black hole quencher BHQ1 at the 3′ end.

**Table 2:** WNV-positive samples analysed by whole genome sequencing.

Sample ID	Sampling Period	Matrix	Ct Value ^1^	Accession No.	% Genome Covered
TI_SUPSI_C28	2–9 August	FTA card	28.6	OR091151	92.69
TI_SUPSI_C37	9–17 August	FTA card	24.2	OR091152	97.58
TI_SUPSI_C65	28 August–6 September	FTA card	27.8	OR091153	95.96
TI_SUPSI_C70	6–12 September	FTA card	28.0	OR091154	98.26
TI_SUPSI_M63	19 July–2 August	*Culex pipiens*	31.5	OR091155	95.83
TI_SUPSI_M158	9–17 August	*Culex pipiens*	29.3	OR091156	96.76
TI_SUPSI_M224	28 August–6 September	*Culex pipiens*	27.9	OR091158	98.23
TI_SUPSI_M202	27 September–5 October	*Culex pipiens*	14	OR091157	99.19

FTA: Flinders Technology Associates. ^1^ Threshold cycle (Ct) values corresponding to the WNV3′ RT-qPCR.

## Data Availability

Genome sequences presented in this study are openly available in GenBank database (accession numbers OR091151–OR091158).
